# Predictive Value of ^18^F-FDG PET/CT-Based Radiomics Model for Occult Axillary Lymph Node Metastasis in Clinically Node-Negative Breast Cancer

**DOI:** 10.3390/diagnostics12040997

**Published:** 2022-04-15

**Authors:** Kun Chen, Guotao Yin, Wengui Xu

**Affiliations:** 1 Department of Molecular Imaging and Nuclear Medicine, Tianjin Medical University Cancer Institute and Hospital, Huanhuxi Road, Hexi Distinct, Tianjin 300060, China; kunchen2022@163.com (K.C.); sxlfygt@sina.com (G.Y.); 2 National Clinical Research Center for Cancer, Tianjin Key Laboratory of Cancer Prevention and Therapy, Tianjin’s Clinical Research Center for China, Tianjin 300060, China

**Keywords:** radiomics, ^18^F-FDG PET/CT, invasive ductal breast cancer, clinically negative axillary lymph node

## Abstract

Background: To develop and validate a radiomics model based on ^18^F-FDG PET/CT images to preoperatively predict occult axillary lymph node (ALN) metastases in patients with invasive ductal breast cancer (IDC) with clinically node-negative (cN0); Methods: A total of 180 patients (mean age, 55 years; range, 31–82 years) with pathologically proven IDC and a preoperative ^18^F-FDG PET/CT scan from January 2013 to January 2021 were included in this retrospective study. According to the intraoperative pathological results of ALN, we divided patients into the true-negative group and ALN occult metastasis group. Radiomics features were extracted from PET/CT images using Pyradiomics implemented in Python, *t*-tests, and LASSO were used to screen the feature, and the random forest (RF), support vector machine (SVM), stochastic gradient descent (SGD), and k-nearest neighbor (KNN) were used to build the prediction models. The best-performing model was further tested by the permutation test; Results: Among the four models, RF had the best prediction results, the AUC range of RF was 0.661–0.929 (mean AUC, 0.817), and the accuracy range was 65.3–93.9% (mean accuracy, 81.2%). The *p*-values of the permutation tests for the RF model with maximum and minimum accuracy were less than 0.01; Conclusions: The developed RF model was able to predict occult ALN metastases in IDC patients based on preoperative ^18^F-FDG PET/CT radiomic features.

## 1. Introduction

Breast cancer is the most common malignant tumor for women, and it is also the second leading cause of death [1,2]. Nearly one-third of breast cancer patients will metastasize to the lymph nodes. Whether ALN metastasis is the important predictor of overall recurrence and the staging of breast cancer [3].

Currently, non-invasive imaging modalities and needle biopsy are mainly used to determine the status of ALN in breast cancer patients before surgery [4]. CN0 was defined by the absence of suspicious nodes on ultrasound imaging and PET/CT, or the absence of tumor cells at fine-needle aspiration [5]. In fact, some cN0 patients have actually developed ALN metastases [6,7]. The treatment regimen and 5-year survival rate of patients with ALN metastases are different from those of patients without lymph node metastases [8]. Sentinel lymph node biopsy (SLNB) and axillary lymph node dissection (ALND) contribute to the diagnosis of false-negative ALN metastases in cN0 patients, but with a certain degree of trauma and some complications [9,10]. Taken together, it is necessary to find a non-invasive and high-accuracy method to further identify occult ALN metastases in cN0 patients.

^18^F-FDG PET/CT is one of the non-invasive imaging modalities and has a wide range of applications in breast cancer diagnosis, staging, and prognosis [11,12,13]. Recently, many researchers have focused on the PET/CT conventional indicators of primary lesion in predicting occult ALN metastasis in cN0, among which SUVmax and TLG have been proved to have a certain significance [14,15]. Radiomics is the high-throughput extraction of image features in the region of interest (ROI) from medical images through computer post-processing technology, and deep mining of biological information contained in images, revealing tumor heterogeneity, genetic characteristics, and others through quantitative analysis phenotypic information [16]. This field is rapidly evolving and is becoming a powerful tool for precision medicine [17]. Recently, several studies have demonstrated the value of radiomics in predicting lymph node metastasis in many malignancies [18], but no further studies have been conducted in breast cancer patients with cN0.

In this study, we aimed to improve the diagnostic performance of occult ALN metastases in IDC patients with cN0 through ^18^F-FDG PET/CT radiomics.

## 2. Materials and Methods

### 2.1. Patients

**Figure 1:**
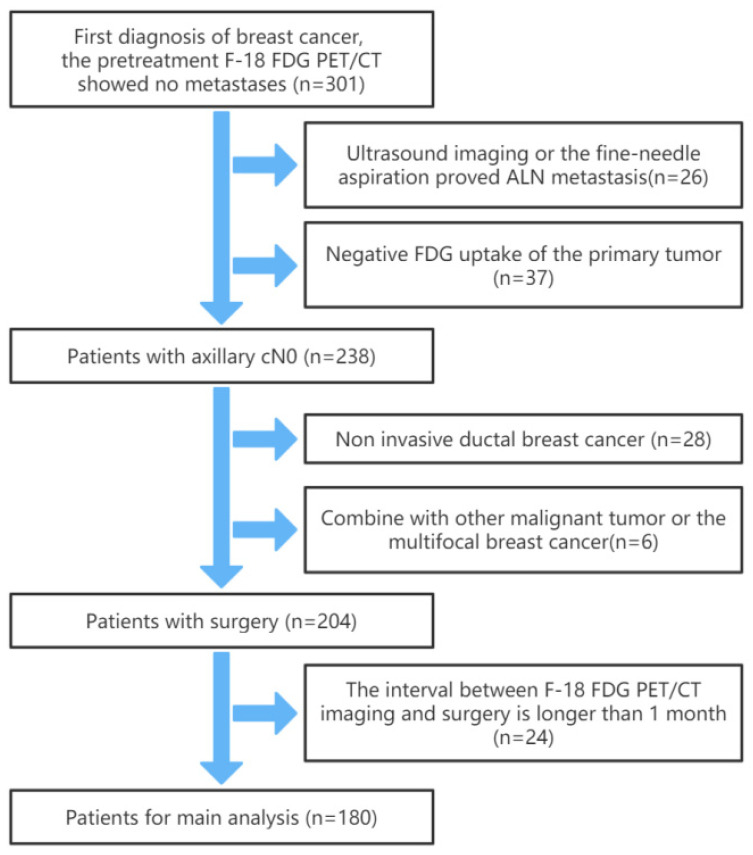
The flowchart of patient selection.

We retrospectively analyzed the clinical and PET/CT data of 180 patients with postoperative pathological diagnosis of breast cancer at Tianjin Medical University Cancer Institute and Hospital from January 2013 to January 2021. All patients underwent surgery and intraoperative SLNB or ALND. The inclusion criteria were as follows: (1) Breast cancer patients who have undergone ^18^F-FDG PET/CT imaging and have been confirmed as cN0 by ultrasound imaging and ^18^F-FDG PET/CT, or the fine-needle aspiration. (2) Postoperative pathologically confirmed IDC. (3) No previous breast surgery, and no breast cancer treatment before ^18^F-FDG PET/CT imaging. (4) All patients had complete clinical data and clear ^18^F-FDG PET/CT images. The exclusion criteria were: (1) Primary tumor ^18^F-FDG PET/CT uptake was negative (SUVmax < 2.5). (2) Postoperative pathologically confirmed multifocal breast cancer or the presence of breast cancer in both breasts. (3) Combined with other malignant tumors in the past. (4) The interval between ^18^F-FDG PET/CT imaging and surgery was longer than 1 month. The flowchart of patient selection is shown in Figure 1. All procedures in studies involving human participants were conducted by the 1964 Helsinki Declaration and its later amendments or comparable ethical standards.

### 2.2. PET/CT Image

#### 2.2.1. Image Acquisition

All patient data were obtained on a PET/CT scanner (GE Discovery Elite). Patients were instructed to fast for at least 6 h before the PET/CT imaging and to control fasting blood glucose concentration to <11.1 mmol/L. ^18^F-FDG (0.10–0.15 mci/kg) was injected through the cubital vein, after which the patient rested in a dark and quiet environment for approximately 60 min (the mean injection–acquisition time delay was 63.7 min; injection-collection time delay range was 50–73 min). All patients were scanned from the base of the skull to the distal femur. The spiral CT scan was performed with a tube current of 180 mAs, a tube voltage of 120 kV, and a layer thickness of 5 mm. The PET was scanned in eight bed positions, and each bed position needs 2 min with increments of 16.2 cm (3D mode). The ordered-subset expectation maximization (OSEM) iterative algorithm (with 6-mm full-width-at-half-maximum Gaussian filter) was used to reconstruct the PET images with CT values being used for attenuation correction, and the final PET voxel size was 5.3 mm × 5.3 mm × 2.5 mm.

#### 2.2.2. PET/CT Image Feature Extraction

**Figure 2:**
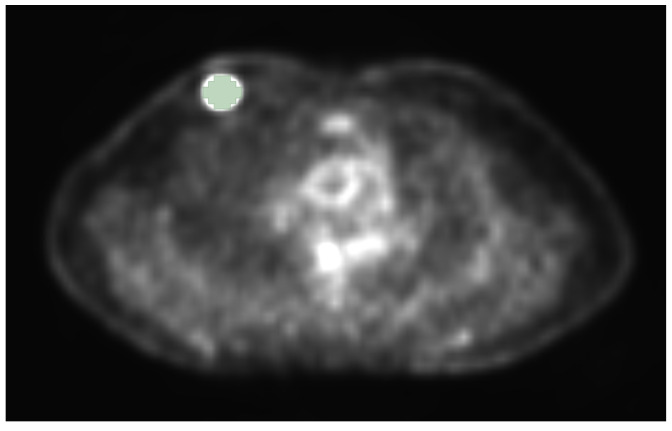
The 3D slicer was used to segment the primary lesions of IDC patients with cN0, and the threshold was SUV = 2.5.

To extract the PET/CT imaging texture features of the lesions, we used 3D Slicer (version: 4.11.20210226) software to manually outline the primary tumor. CT and PET images in the DICOM format were imported into 3D Slicer and the lesions were delineated slice by slice. PET images with standardized uptake value (SUV = 2.5) as the threshold to outline the region of interest (ROI), as shown in Figure 2. In order to reduce inter-individual variability, two nuclear medicine physicians segmented the images without knowing the results, and one of the doctors segmented the image twice. Intraclass correlation coefficients (ICCs) were used to evaluate the repeatability of radiomics features extraction within and among observers. An ICC of >0.75 is considered to have good reliability.

**Figure 3:**
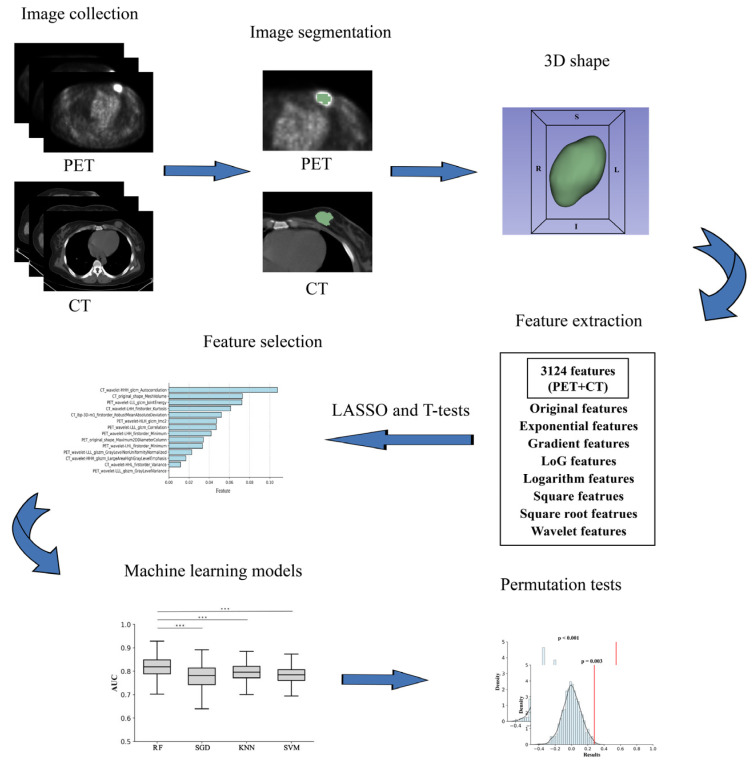
The workflow for image segmentation, feature extraction, feature selection, machine learning models and permutation tests.

Then, we resampled the VOIs of CT and PET images to unify them to an isotropic voxel size of 1.0 × 1.0 × 1.0 mm^3^. Using the Python software and the Pyradiomics module, the radiomics features within the VOI of the resampled PET and CT images were extracted, respectively. Features include first order, shape (2D), shape (3D), gray level co-occurrence matrix (GLCM), gray level size zone matrix (GLSZM), gray level run length matrix (GLRLM), neighboring gray tone difference matrix (NGTDM), and gray level dependence matrix (GLDM). A total of 1562 PET features and 1562 CT features were extracted, and 3124 PET/CT features were obtained by integrating the results. The workflow for image segmentation, feature extraction, feature selection, and machine learning models establishment and permutation tests is shown in Figure 3.

### 2.3. Definition of ALN Metastasis Negative

Through PET/CT images, we often refer to the morphology criteria and the maximum SUV (SUVmax) of the lesions to judge the biological behavior of the tumor. SUVmax was calculated using the following formula: SUVmax = maximum activity in a region of interest (MBq/g)/[injected dose (MBq)/body weight (g)]. Regarding the condition of ALN: (1) SUVmax ≥ 2; (2) Short diameter > 0.5 cm, rounded shape, eccentric cortical hypertrophy, and absence of a fatty hilum for morphology. If neither of these points is satisfied, it is defined as ALN metastasis negative.

### 2.4. Statistical Analysis

We employed *t*-tests and LASSO to eliminate uncorrelated and redundant features and improve the classification performance of the classifier. Relevant features were constructed with four classifiers: RF, SVM, SGD and KNN in the skelearn module. Considering the unbalanced ratio of ALN metastasis and true negatives (1:5.4) in cN0 patients, we performed an under-sampling method to analyze the results of the two groups. The specific method was as follows: We randomly selected 28 individuals from 152 true negatives in cN0 to ensure that the number of true negative patients in cN0 was the same as the number of ALN metastasis patients. Then, the four classifier models mentioned above were built. This process was repeated 1000 times to eliminate the effect of sampling randomness [19]. We performed the independent samples *t*-tests for precision and AUC for all models, and permutation tests for the highest and lowest precision of the best performing model. Analysis of clinical characteristics with a *t*-test and Chi-square test. All graphics are drawn by python (version: 3.7.9). A *p*-value of <0.05 was defined as statistical significance.

## 3. Results

### 3.1. Clinical Characteristics

The characteristics of the patients who participated in the study are shown in Table 1. We included 180 patients with pathological results of IDC and a clinical diagnosis of cN0. According to the results of SLNB or ALND, we confirmed that 152 patients (mean age, 56 years; range, 31–82 years) had no ALN metastasis, and 28 patients (mean age, 55 years; range, 38–76 years) had ALN metastasis. There were no significant differences in clinical characteristics between the two groups (*p* > 0.05). The analysis found that the ICCs between the measurements of the first radiologist was 0.794 to 0.986, and between the measurements of two radiologists were 0.761 and 0.972. Since both within-group and between-group consistency were >0.75, data from the first radiologist were used for subsequent analysis.

### 3.2. Feature Extraction and Model Comparison

**Figure 4:**
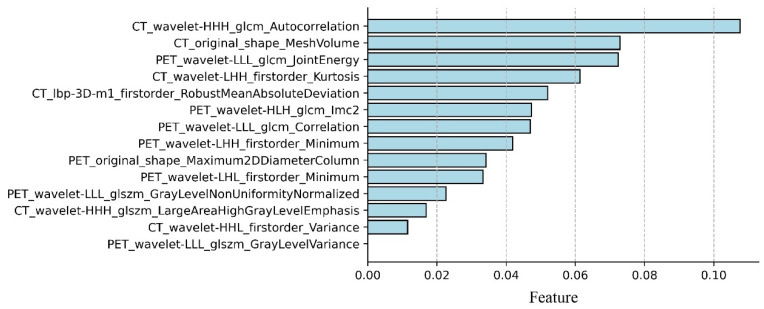
Important parameters and weights for predicting occult ALN metastasis in IDC patients with cN0 by *t*-test and LASSO screening.

A total of 3124 radiomic features were extracted for each patient. The features were screened by *t*-test and LASSO, and 14 radiomic features that were important for predicting ALN metastasis in breast cancer cN0 patients were obtained, including eight PET features and six CT features. Figure 4 shows important radiomic features predicting ALN metastasis in IDC patients with cN0, with CT_wavelet-HHH_glcm_Autocorrelation having the highest signature coefficient.

**Figure 5:**
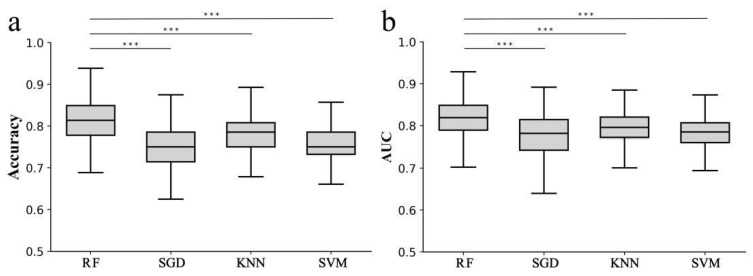
Distribution and comparison of accuracy and AUC of RF, SGD, KNN and SVM. (**a**) distribution and comparison of accuracy of RF (quartile: 81.4%; mean: 81.2%), SGD (quartile: 75.0%; mean: 74.5%), KNN (quartile: 78.6%; mean: 78.5%) and SVM (quartile: 75.0%; mean: 75.6%). The accuracy of RF was higher than SGD, KNN and SVM, and the effect is remarkable (*p* < 0.001). (**b**) distribution and comparison of AUC of RF (quartile: 0.819; mean: 0.817), SGD (quartile: 0.782; mean: 0.775), KNN (quartile: 0.796; mean: 0.795) and SVM (quartile: 0.785; mean: 0.783). The AUC of RF was higher than SGD, KNN and SVM, and the effect is remarkable (*p* < 0.001). *** *p*-value < 0.001.

Then, four models were established according to the screened important features. For every kind of model, under-sampling was performed 1000 times. The AUC range of RF was 0.661–0.929 (mean AUC, 0.817), the accuracy range was 65.3–93.9% (mean accuracy, 81.2%); the AUC range of SGD was 0.506–0.892 (mean AUC, 0.775), the accuracy range was 50.0–87.5% (mean accuracy, 74.5%); the AUC range of KNN was 0.645–0.885 (mean AUC, 0.795), the accuracy range was 64.3–89.3% (mean accuracy, 78.5%); the AUC range of SVM was 0.660–0.877 (mean AUC, 0.783) and the accuracy range was 60.7–89.3% (mean accuracy, 75.6%). The comparison of AUC and the accuracy of the four models are shown in Figure 5. RF was considered the best model, with *p* < 0.001 compared to SGD, KNN and SVM.

### 3.3. Model Validation

**Figure 6:**
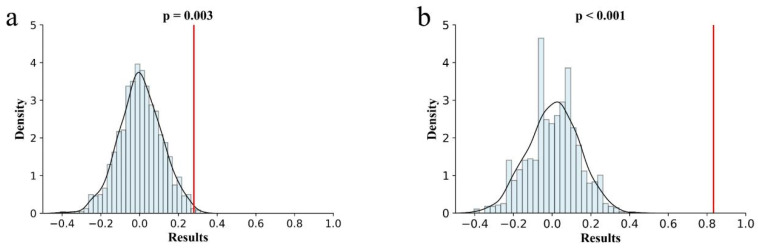
Permutation tests for RF with the highest and lowest accuracy. (**a**) permutation tests for RF with the lowest accuracy (65.3%, *p* = 0.003 < 0.01, Sobs = 0.280); (**b**) permutation tests for RF with the highest accuracy (93.9%, *p* < 0.001, Sobs = 0.833).

In order to verify the effect of the optimal RF model established with the small sample size, we performed a permutation test on the groups with the highest and lowest accuracy of the RF model, and the results are shown in Figure 6. The permutation test for the minimum precision had a Sobs value of 0.280 and the *p*-value < 0.01; the maximum precision of the Sobs value was 0.833 and the *p*-value < 0.001.

## 4. Discussion

The status of ALN in breast cancer patients affects the choice of treatment and prognosis. In addition, 15–20% of patients with the clinical diagnosis of cN0 still have ALN metastases [6,7,20], but there is currently no particularly effective method to further judge the status of ALN in patients with cN0 breast cancer before surgery. SLNB and ALND are currently the surgical methods for accurate staging of the axilla in cN0 patients, but they are accompanied by many adverse reactions, including arm lymphedema, paresthesia, and shoulder dysfunction or numbness [21,22]. In this study, we constructed four models from the screened important parameters to predict the ALN status of breast cancer cN0 patients, respectively. Among them, the prediction effect of the RF model is better than SGD, KNN and SVM.

The preoperative diagnosis of occult ALN metastases in cN0 breast cancer patients is constantly being studied. Merrill et al. found that routine evaluation with complete blood count, liver function tests, alkaline phosphatase tests and chest X-ray was low yield in cN0 invasive breast cancer patients, and there was an inability to distinguish occult ALN metastases [23]. ^18^F-FDG PET/CT is one of the important examination methods for the diagnosis of breast cancer cN0. Its advantages are not only systemic examination but also provide information on the anatomy and function. Yoo et al. demonstrated that TLG of primary tumors can be useful in predicting ALN metastasis in IDC patients with cN0, and when the cutoff value of TLG is 5.74, the maximum AUC value is 0.794 (95% CI 0.716–0.859) [15]. Yamagishi et al. performed two ^18^F-FDG PET/CT images of breast cancer patients at 60 min (SUVmax1) and 120 min (SUVmax2) and calculated ΔSUVmax% by formula ΔSUVmax% = [(SUVmax2 − SUVmax1)/SUVmax1] × 100. They found that the combination of SUVmax2 and ΔSUVmax% may be useful for predicting non-sentinel node metastases in the ALN of cN0 patients [14]. In this study, we enriched the features obtained from ^18^F-FDG PET/CT with radiomics, including not only conventional parameters such as SUVmax, original shape, but also gray-scale patterns, spectral properties, and inter-pixel relationships of clinical images. All important features after dimensionality reduction were applied to the four different models, and the best prediction model was derived by combining AUC and accuracy. On the basis of previous related studies, we obtained as much information as possible from PET/CT and established more complex models, which further improved the predictive effect of PET/CT on IDC patients with cN0 to a certain extent.

Radiomics is a technology that uses high-throughput feature extraction technology to extract and quantify deep information in image data, and finally use it for decision-making [24,25,26]. It can extract shape features, first-order features and high-order features of images, some of which have the potential to evaluate patient diagnosis and prognosis [27]. Therefore, some scholars applied radiomics in the study of malignant tumor patients with cN0. Wang et al. developed the diagnostic PET/CT-based radiomics nomogram, including CEA, MTV, and the radiomics signature to predict the OLM in cN0 solid lung adenocarcinoma, and demonstrated adequate discrimination in the training set (C-index, 0.769) and the validation set (C-index, 0.768) [18]. Wu et al. developed a radiomics nomogram for non-invasive preoperative prediction, which combines radiomics features with CT-reported LN status, with good predictive accuracy for lymph node metastasis in bladder cancer patients, especially for cN0 patients, the study showed good calibration and discrimination in the training set [AUC, 0.9262; 95% confidence interval (CI), 0.8657–0.9868] and the validation set (AUC, 0.8986; 95% CI, 0.7613–0.9901) [28]. It is necessary to determine the actual ALN status of IDC preoperatively, but there is currently no application of radiomics in this field. In this study, the top three features of normalized data weights were CT_wavelet-HHH_glcm_Autocorrelation, CT_original_shape_MeshVolume, and PET_wavelet-LLL_glcm_JointEnergy, where the PET_wavelet-LLL_glcm_JointEnergy feature was consistent with Song’s study of predicting ALN metastasis using radiomics [29]. This indicates that PET_wavelet-LLL_glcm_JointEnergy plays an important role in predicting both occult ALN metastasis with cN0 and ALN metastasis in IDC patients. To account for the reliability of the model established with a small amount of data after under-sampling, we performed a permutation test on the highest and lowest accuracy of the RF model, further confirming that the model is a reliable method for noninvasively predicting ALN metastasis in IDC patients with cN0.

The short diameter of 5 mm and SUVmax 2.0 was used as the judgment indicators of ALN status in this study. Shien et al. and Ogino et al. were in agreement that the best sensitivity and specificity results were achieved when the short diameter cutoff value was 5 mm [30,31]. Carkaci et al. compared the diagnostic performance of SUV values of 2.0, 2.5, and 3.0 and concluded that the SUVmax cutoff 2.0 provided the highest diagnostic accuracy [32]. For the extraction of the primary lesion ROI, we manually segmented the image using SUV 2.5 as the threshold [33,34]. Although the ROI cannot contain the lesion with an SUV value lower than 2.5, it can ensure a good segmentation effect while ensuring reproducibility. This method has been widely used in the segmentation of breast cancer primary lesions.

Previous studies reported that the clinical outcomes of breast cancer were correlated with several clinicopathological parameters. Among them, SUV is the most commonly used semi-quantitative analysis index in ^18^F-FDG PET/CT imaging, and is related to tumor cell proliferation and malignancy, and ki-67 is also related to tumor cell proliferation. Furthermore, a variety of studies have shown a significant correlation between ki-67 and SUV [35,36]. However, in this study, neither SUVmax nor ki-67 was able to effectively differentiate the two groups. Presumably, the occurrence of occult lymph nodes metastasis in breast cancer patients with cN0 is not closely related to the malignancy of tumor cells. Additionally, the molecular subtype classification for breast cancer was also a commonly accepted index influencing the clinical outcome of breast cancer, whereas, as shown in the result, the discrimination of the two groups was not remarkably related to the molecular subtype of breast cancer. The lymph node status of breast cancer patients is one of the important reasons that affects the selection of treatment modality, and also affects the formulation of neoadjuvant therapy [37]. As the first stop of lymph node metastasis, it is important to determine the true status of axillary lymph nodes in cN0 patients before surgery, but there is currently no effective method. Our study can not only provide a reference to predict the real axillary lymph node status before surgery for breast cancer with cN0, but also provide an innovative clue for the research of occult lymph node metastasis in breast cancer.

There were still some limitations in our study. First, this prediction method is currently only validated in a single center and requires a standardized method of data from different devices to generalize the validation method and to obtain external validation. In addition, the number of enrolled patients was relatively small. Second, we regarded the pathological results of SLNB and ALND as the actual state of ALN. Considering that SLNB has a lower probability of complications than ALND, and SLNB-negative patients have no significant difference from ALND in terms of disease-free survival (DFS), overall survival (OS), and moreover regional node recurrence event, SLNB-negative patients do not undergo complete ALND anymore. However, there is a false-negative rate of 4.6–16.7% with an average of 8.4% for SLNB, which could have some impact on the results of this study [38,39]. Finally, neoadjuvant chemotherapy (NAC) affects the ALN status of IDC patients with cN0 [40], but those who experienced NAC before surgery were excluded from this study. It is necessary for these patients to be further studied to verify the generalizability of the prediction method of this study.

## 5. Conclusions

In this study, we proposed a quantitative prediction model for PET/CT images based on radiomics, which extracted multimodal and multidimensional features by *t*-tests and LASSO, and combined the results of multiple classifiers for comparison. Among all models, the RF model had the best prediction of ALN status for IDC breast cancer with cN0, with the AUC range of RF being 0.661–0.929 (mean AUC, 0.817) and the accuracy range being 65.3–93.9% (mean accuracy, 81.2%). This RF-based predictive model may partially help clinicians to determine the ALN status of IDC with cN0 preoperatively by non-invasive imaging.

## Figures and Tables

**Table 1:** Patient characteristics.

Patient Characteristics	Breast Cancer (cN0)		*p*
	ALN Metastasis (+) (*n* = 28)	ALN Metastasis (−) (*n* = 152)	
Age (mean ± SD, years)	55.93 ± 12.40	54.99 ± 12.28	0.713
Menopausal state			0.749
Pre	11 (39.3%)	53 (34.9%)	
Peri	4 (14.3%)	17 (11.2%)	
Post	13 (46.4%)	82 (53.9%)	
ER status			0.960
Negative	5 (17.9%)	31 (20.4%)	
Positive	23 (82.1%)	121 (79.6%)	
PR			0.912
Negative	6 (21.4%)	28 (18.4%)	
Positive	22 (78.6%)	124 (81.6%)	
Ki-67 status			0.871
Negative (<14%)	8 (28.6%)	38 (25.0%)	
Positive (≥14%)	20 (71.4%)	114 (75.0%)	
Molecular subtypes			0.965
Luminal A	7 (25.0%)	33 (21.7%)	
Luminal B (HER2 negative)	5 (17.9%)	28 (18.4%)	
Luminal B (HER2 positive)	7 (25.0%)	32 (21.1%)	
HER2 positive	5 (17.9%)	34 (22.4%)	
Triple-negative	4 (14.2%)	25 (16.4%)	
SUVmax	6.68 ± 2.69	6.31 ± 2.77	0.513

## Data Availability

The data that support the findings of this study are available on request from the corresponding author. The data are not publicly available due to privacy or ethical restrictions.
